# SIRT3 Regulation Under Cellular Stress: Making Sense of the Ups and Downs

**DOI:** 10.3389/fnins.2018.00799

**Published:** 2018-11-02

**Authors:** Joshua M. Marcus, Shaida A. Andrabi

**Affiliations:** ^1^Departments of Pharmacology and Toxicology, The University of Alabama at Birmingham, Birmingham, AL, United States; ^2^Department of Neurology, The University of Alabama at Birmingham, Birmingham, AL, United States

**Keywords:** SIRT3, stroke, mitochondria, bioenergetics, NAD+, PARP

## Abstract

Sirtuin 3 (SIRT3) is an NAD+ dependent deacetylase that resides primarily in mitochondria and functions to maintain mitochondrial homeostasis under stress. SIRT3 expression has been observed to change under a number of different stresses in multiple tissues and model systems. Inconsistencies in the literature with regards to how and when SIRT3 protein levels change indicates that the mechanism of SIRT3 regulation is multi-faceted. Alterations in SIRT3 have been observed in experimental models of cellular stress, however, the effect these changes have on mitochondrial health remain unknown. Neurons are highly dependent on proper mitochondrial function for their survival. SIRT3 dynamics and function have been studied using models of genotoxic, metabolic, and oxidative stresses, although it remains unclear how SIRT3 is being regulated under these conditions. A closer look into SIRT3 regulation under stress conditions in various model systems will help incorporate the many SIRT3 regulatory mechanisms at play in disease states. In this review, we describe the observations that have been made about SIRT3 protein modulation under basic stress conditions. We then point out consistencies and contradictions in these observations and what they mean. Lastly, we present the observations made in the complicated neuronal stress of stroke. We hope that this review will help consolidate the ambiguous SIRT3 literature and provide a framework for investigation of SIRT3 regulation during stress response.

## Introduction: SIRT3 Function and Tissue Specific Expression

In mammalian systems, SIRT3 is one of seven homologs of the yeast histone deacetylase, silent information regulator (Sir2) (Frye, 1999, 2000). These homologs comprise a family of NAD+ dependent post-translational modifying proteins, all of which have different enzymatic activity and subcellular localization. SIRT3, along with SIRT4 and SIRT5 are predominantly localized to mitochondria, although SIRT3 has been reported to have additional function in the cytosol and nucleus (Michishita et al., 2005; Sundaresan et al., 2008; Huang et al., 2010). Knockout studies of SIRT4 and SIRT5 result in minimal changes in mitochondrial protein acetylation, while SIRT3 knockout results in significantly increased acetylation of mitochondrial and non-mitochondrial proteins (Lombard et al., 2007; Finkel et al., 2009). This led researchers to believe that SIRT3 is the major deacetylase in mitochondria. Human SIRT3 is found in two isoforms, a full-length 44 kDa form which is cleaved within mitochondria by matrix metalloprotease to a 28 kDa short form (Schwer et al., 2002). The long isoform has been found in mitochondria, cytoplasm, and nucleus, and the short isoform localizes to mitochondria (Scher et al., 2007; Iwahara et al., 2012). It is believed that only the 28 kDa isoform is an active NAD+ dependent deacetylase, although the deacetylase activity of full length SIRT3, particularly its ability to act as a histone deacetylase in the nucleus is controversial (Hallows et al., 2008).

About one fifth of all mitochondrial proteins are targets for deacetylation by SIRT3 (Hebert et al., 2013). Among these proteins include components of oxidative phosphorylation, fatty acid oxidation, tricarboxylic acid (TCA) cycle, and amino acid metabolism. The dependence of SIRT3 on regulating the activity of such proteins as acetyl-CoA-synthetase 2, glutamate dehydrogenase, and the complex I subunit NDUFA9 are among the first to be characterized (Hallows et al., 2006; Lombard et al., 2007; Ahn et al., 2008). Human Acetyl-CoA Synthetase 2 (AceCS2) has a lysine acetylation site at Lye-642 located in the catalytic site. Deacetylation of this residue by SIRT3 directly activates AceCS2 through this mechanism (Schwer et al., 2006). Similar activation mechanisms have been proposed for the other SIRT3 target enzymes, including isocitrate dehydrogenase 2 and long-chain acyl CoA dehydrogenase (Schlicker et al., 2008; Someya et al., 2010). More recently, proteomic approaches have been applied to identify targets for deacetylation, as well as potential protein binding partners (Sol et al., 2012; Yang et al., 2016). Key observations from these data suggest that fatty acid metabolism and the TCA cycle are among the most heavily regulated by SIRT3.

SIRT3 is highly expressed in tissues with high metabolic demand. These include, cardiac muscle, liver, kidney, brown adipose tissue, and skeletal muscle (Jin et al., 2009). These tissues are also more exposed to stress conditions such as hypoxia and oxidative stress that disrupt mitochondrial function. Early studies using SIRT3 knockout mice revealed no observable developmental defects. It was only after knockout animals were metabolically challenged that phenotypic differences could be observed (Hirschey et al., 2010). This suggests that SIRT3 has a more prominent role in maintaining mitochondrial homeostasis after stress than contributing to overall mitochondrial function in metabolically demanding tissue under normal physiological conditions.

There is now a large body of literature describing SIRT3 modulation after stress responses in different tissue types and model systems, and at first glance these results seem inconsistent. Investigators have reported up regulation and down regulation of both proteolytically cleaved active SIRT3 and the full-length isoform, sometimes within the same experimental paradigm, and there does not seem to be a clear and logical explanation. There are also reports of translocations that are contended by various groups (Hallows et al., 2008; Iwahara et al., 2012). Here, we organize these findings by the type of stress applied and the model system used in order to make sense of the ambiguous results available. We intend to organize the literature that describes SIRT3 dynamics under stress conditions to help investigators make sense of their findings regarding SIRT3 expression under pathological conditions and disease states. We include conditions of genotoxic stress, metabolic stress, and oxidative stress, as well as the complex disease model of stroke which has components of all three. This review serves to demonstrate the often-unintuitive nature of SIRT3 regulation and how it can be applied toward investigating disease pathology.

## SIRT3 Modulation After Genotoxic Stress

Numerous reports of SIRT3 protein modulation as a result of genotoxic stress have been published, most of which convey consistent results. *In vitro* models of human kidney cells (HK-2) and multiple hepatocellular carcinoma cell lines show a decrease in SIRT3 protein after treatment with the platinum-based DNA intercalating agent cisplatin (Tao et al., 2016; Yoon and Kim, 2016). In animal models, a decrease in SIRT3 mRNA was observed in kidneys of mice treated with cisplatin (Morigi et al., 2015), and a decrease in SIRT3 protein was observed in kidney cells isolated from cisplatin treated rats (Ugur et al., 2015). Doxorubicin, a DNA intercalating agent, has also been reported to down regulate SIRT3 protein levels in hepatocellular carcinoma cell lines, rat primary cardiomyocytes, and mouse heart lysates (Pillai et al., 2016, 2017; Tao et al., 2016). Model specific differences in SIRT3 modulation are observed after ionizing radiation. One group observed a decrease in SIRT3 in murine lung tissue, while another observed an increase in SIRT3 protein in multiple cancer cell lines, including HCT116, U87, and MDA231, after 5 Gy ionizing radiation treatment *in vitro* (Liu et al., 2015; Cao et al., 2017). This discrepancy could be a result of the difference in cell type or *in vivo* vs. *in vitro* systems.

One possible explanation for the down regulation of SIRT3 observed after cisplatin treatment is through activation of poly(ADP-ribose) polymerases (PARPs). In response to DNA damage, PARPs are activated and synthesize poly(ADP-ribose) polymer (PAR), which bind proteins through ionic and covalent interactions (Gagne et al., 2008). This functions to signal and recruit DNA damage response proteins to sites of DNA strand breaks. Under conditions of severe DNA damage, like after cisplatin treatment, PARPs becomes hyperactivated and PAR polymer accumulates in the cell, leading to cell death (Andrabi et al., 2006; Yu et al., 2006). This phenomenon has been observed to occur in stroke and is thought to be a major contributor to neuron loss (Baxter et al., 2014). Many E3 ligases contain PAR polymer binding sites and become activated upon PAR binding (Andrabi et al., 2011; Kang et al., 2011), which may then target SIRT3 for proteasomal degradation. A previous study demonstrated that the down regulation of SIRT3 in renal cells after cisplatin can be rescued by co-treatment with a PARP inhibitor (Yoon and Kim, 2016). Further experiments are needed to identify whether any specific PAR-dependent E3 ligases facilitate the degradation of SIRT3 under these conditions.

Apart from few reports that demonstrate SIRT3 activity in the nucleus, SIRT3 functions as a deacetylase primarily in the mitochondria, and it unclear whether PARP can regulate SIRT3 activity at the level of substrate concentration. The mitochondrial NAD+ pool is thought to be separate from the cytosolic and nuclear pools (Stein and Imai, 2012; VanLinden et al., 2015), however, this idea is purely speculative due to the inability of NAD+ to defuse through the inner and outer mitochondrial membranes and the lack of a mitochondrial NAD+ transporter found in eukaryotes (Stein and Imai, 2012).

Additionally, the affinities of SIRT3 and PARPs for NAD+ can provide clues as to how local NAD+ concentrations affect enzymatic activity. The Km of SIRT3 for NAD+ was measured to be 880 μM (Hirschey et al., 2011), while the Km of PARP1 for NAD+ is between 30 and 60 μM (Canto et al., 2013). Cellular NAD+ concentrations are estimated to be between 400 and 700 μM (Revollo et al., 2004), suggesting that changes in NAD+ will affect SIRT3 activity but not PARP1 activity. It is still unclear to what extent PARP hyperactivation alters mitochondrial NAD+ concentrations, but even minor changes in mitochondrial NAD+ is likely to affect SIRT3 activity. Studying compartmental NAD+ concentration dynamics has been technically challenging. NADH autofluorescence can be utilized to measure total cellular NAD+/NADH ratios at the single cell level, but this does not address compartmental dynamics (Blacker et al., 2014). Newly developed genetically encoded fluorescent biosensors can be targeted to cellular compartments, such as the nucleus, cytosol or mitochondria, and have been used to measure NAD+/NADH ratios *in vitro* as well as *in vivo* under various conditions (Hung et al., 2011). Future studies using these fluorescent biosensors would determine if mitochondrial NAD+ concentration decreases upon PARP over activation, thereby altering SIRT3 activity.

## SIRT3 Modulation After Metabolic Stress

SIRT3 is an important regulator of mitochondrial function and can up regulate fatty acid oxidation as an adaptive response to metabolic stress such as fasting or caloric restriction. *In vivo*, fasting of mice for 24 h showed an increase in SIRT3 expression in extensor digitorum longus muscle (Gudiksen and Pilegaard, 2017). A separate study reported up regulation of SIRT3 in mouse liver under the same conditions (Li et al., 2016). Up regulation of SIRT3 was observed in murine liver tissue, skeletal muscle, cardiac muscle, brown and white adipose tissue after caloric restriction (Shi et al., 2005; Hirschey et al., 2010, 2011; Jing et al., 2011; Li et al., 2016; Barger et al., 2017; Gudiksen and Pilegaard, 2017; Wang et al., 2017). A comparable *in vitro* model of serum starvation showed an increase in SIRT3 expression in primary murine bone marrow derived mesenchymal stem cells (Wang et al., 2017). One group purposed that after fasting or caloric restriction mitochondrial proteins become acetylated through non-enzymatic mechanisms, rendering these proteins inactivate. In this case, SIRT3 could be up regulated in order to repair acetylated mitochondrial proteins and restore their function (Weinert et al., 2015). These findings suggest that SIRT3 is upregulated in response to nutrient deprivation and helps to restore proper mitochondrial function under these conditions.

Nutrient abundance can also be a form of metabolic stress, and SIRT3 is consistently downregulated under these conditions. Mice given a high fat diet for extended periods of time show a decrease in both protein and transcript levels in skeletal muscle and liver tissue (Hirschey et al., 2011; Pillai et al., 2017). Interestingly, initial administration of a high fat diet leads to increase SIRT3 protein and transcript levels that gradually decrease over time, according to one report (Hirschey et al., 2011). High glucose and insulin treatment is consistently correlated with decreased SIRT3 protein and mRNA in retinal endothelial cells, which may contribute to vision loss associated with hypoglycemia (Hirschey et al., 2011). Streptozotocin induced diabetic mice also show decreased SIRT3 protein and mRNA levels (Jing et al., 2011).

For metabolic stress due to prolonged fasting, data obtained from these studies are inconsistent in the literature in terms of SIRT3 dynamics. Several reports show an increase in SIRT3 in hind limb muscles, and liver after fasting (Hirschey et al., 2010; Li et al., 2016; Gudiksen and Pilegaard, 2017). Others report a decrease in SIRT3 (Jing et al., 2011, 2013; Edgett et al., 2016). Two of these reports were very similar, using the same muscles from C57/Bl6 mice after the same 24 h fasting period and found conflicting results. The one key difference between these studies is the age of the mice used in the study. One group used 3-month-old mice for their experiments and found a significant increase in SIRT3 after 24 h of fasting. Another used 8-week-old mice for their experiment and found a significant decrease in SIRT3 protein and mRNA. SIRT3 is known to affect aging, and overexpression of SIRT3 orthologs has been shown to increase lifespan in C. elegans, Drosophila, and yeast (Guarente, 2007). Polymorphisms associated with increase expression of SIRT3 in humans has also been linking to increased longevity (Albani et al., 2014). With a clear connection between SIRT3 and age associated stress, age could be a confounding factor in SIRT3 expression and activity regulation, and this could explain the inconsistency in published data. However, further understanding of SIRT3 regulation is required to reveal the biological differences in these experiments.

One explanation for changes in SIRT3 expression under metabolic stress is Keap1/Nrf2/ARE signaling. Antioxidant response elements (ARE) can be found upstream of stress response genes, which include heat shock proteins, proteins involved in maintaining redox homeostasis, and sirtuins (Wang et al., 2007; Calabrese et al., 2010). Indeed, SIRT3 contains an upstream ARE where the transcription factor nuclear factor erythroid 2-related factor (Nrf2) binds (Satterstrom et al., 2015). Under basal conditions, ARE binding by Nrf2 is repressed by Kelch-like ECH-associated protein 1 (Keap1), which binds Nrf2, prevents its nuclear translocation, and facilitates is proteasomal degradation (Taguchi et al., 2011). The human isoform of Keap1 contains cysteine residues which are susceptible to oxidative damage, making Keap1 function as a redox sensor (Taguchi et al., 2011). Under stress conditions where reactive oxygen species accumulate in the cell, conserved cysteine residues of Keap1 become damaged, which induces a conformational change in the protein and abolishes Keap1-Nrf2 binding in the cytoplasm. Nrf2 is then free to translocate into the nucleus and facilitate transcription of SIRT3, as well as numerous other stress-response and antioxidant genes (Calabrese et al., 2011).

## SIRT3 Modulation After Oxidative Stress

Oxidative stress due to oxygen deprivation or treatment with hydrogen peroxide lead to mitochondrial dysfunction, and SIRT3 may be important for regulating enzymatic activity under these conditions. There are multiple reports of SIRT3 modulation after induced oxidative stress in the literature. Some inconsistencies are present, but this may be due to the complexity of cellular processes that are affected by oxidative stress, such as DNA damage, metabolic shifts, and cellular stress responses, all of which have been shown to affect SIRT3 protein and transcript levels.

Human tissue, mice, and rats exposed to extreme hypoxic conditions show effects on SIRT3 expression dynamics, but the mechanism of this modulation is not clear. Several studies report a decrease in SIRT3 protein levels. These include primary mouse cardiomyocytes, the mouse hippocampal cell line HT-22, cortex from rats exposed to a hypoxic environment, and human visceral adipose tissue explant cultures (Garcia-Fuentes et al., 2015; Yang et al., 2015; Liu et al., 2016; Zhang et al., 2017). An increase in SIRT3 protein and mRNA was observed in human umbilical vein endothelial cells (HUVECs) exposed to a 2% hypoxic condition (Tseng et al., 2014). Human glioma cell line LN229 also showed an increase in SIRT3 protein after hypoxic treatment (Qiao et al., 2016). The latter two examples could represent model specific differences in SIRT3 regulation.

Hydrogen peroxide treatment is also poorly understood in terms of how it affects SIRT3 dynamics. One study found a decrease in SIRT3 protein levels in both mouse and human derived alveolar epithelial cells (Jablonski et al., 2017). HUVECs saw an increase in SIRT3 protein after Hydrogen peroxide treatment, and primary neonatal rat heart myocytes saw an increase in only the 44 kDa form of SIRT3.

## SIRT3 Modulation After Stroke

The pathological condition of stroke involves both metabolic and hypoxic stress, and under these conditions, SIRT3 dynamics are more difficult to explain. Similar to starvation and caloric restriction conditions, SIRT3 upregulation is observed after oxygen and glucose deprivation in rat cortical neurons, differentiated PC12 cells, and co-cultures of microvascular endothelial cells and astrocytes (Wang et al., 2015; Dai et al., 2017; Chen et al., 2018). In primary cultures of rat spinal cord neurons, however, investigators found that SIRT3 protein and mRNA levels decrease (Liu et al., 2017). *In vivo* models of stroke reveal increase SIRT3 activity, as determined by a decrease in protein acetylation, but no significant change in SIRT3 protein level (Verma et al., 2018). This suggests that total SIRT3 expression is unchanged after stroke, but its activity is regulated through a mechanism yet to be determined.

Numerous reports link SIRT3 to mitochondrial function after oxygen and glucose deprivation *in vitro* or *in vivo* models of ischemic stroke, which is consistent with increased activity. One such report found deacetylation of ceramide synthases by SIRT3 functions to increase the activity of ceramide synthase isoforms CerS1, CerS2, and CerS6 (Novgorodov and Gudz, 2011). Increase ceramide production in mitochondria is linked to mitochondrial dysfunction and cell death, and this could represent an important mechanism of mitochondrial dysfunction in ischemic stroke (Novgorodov and Gudz, 2011). They also report smaller infarct volume in SIRT3 KO mouse brain after middle cerebral artery occlusion (MCAO) compared to WT. They hypothesize that protection in SIRT3 KO mice is due to decrease ceramide synthesis via hyper-acetylation of ceramide synthases (Novgorodov et al., 2016). Another report found that SIRT3 was responsible for the neuroprotective effects of ketone treatment following cerebral ischemia (Yin et al., 2015). SIRT3 was found to be involved in inhibiting astrocyte activation in a mouse MCAO model which decreased glial scarring (Yang et al., 2017). It has been purposed that SIRT3 activity could be regulated by mitochondrial uncoupling protein 2 (UCP2) during cerebral ischemia-reperfusion injury by increasing the available NAD+/NADH ratio (Su et al., 2017). Together, these findings demonstrate that stroke modulates SIRT3 expression and activity, although the precise mechanism remains elusive.

PARP activation, a major component of stroke pathology (Komjati et al., 2005) could play an important role in SIRT3 regulation. As previously stated, PARP hyper-activation results in accumulation of PAR in the cell, which can interact with numerous proteins (Gagne et al., 2008), potentially resulting in conformational changes that may alter the protein’s localization and function (Leung, 2014). PAR binding to proteins negatively impacts mitochondrial and bioenergetics function (Baek et al., 2013; Andrabi et al., 2014). Under conditions like caloric restriction, SIRT3 activity helps to prevent damage to the mitochondria. However, during stroke when mitochondria are damaged, SIRT3 activity could be detrimental and could exacerbate mitochondrial damage. Indeed, PARP inhibitors have proven effective in preventing mitochondrial damage after stroke. Further experiments that address SIRT3 activity when PARP is inhibited in stroke models would uncover whether PAR dependent mitochondrial damage is exacerbated by SIRT3 activity in stroke.

## Conclusion

**FIGURE 1:**
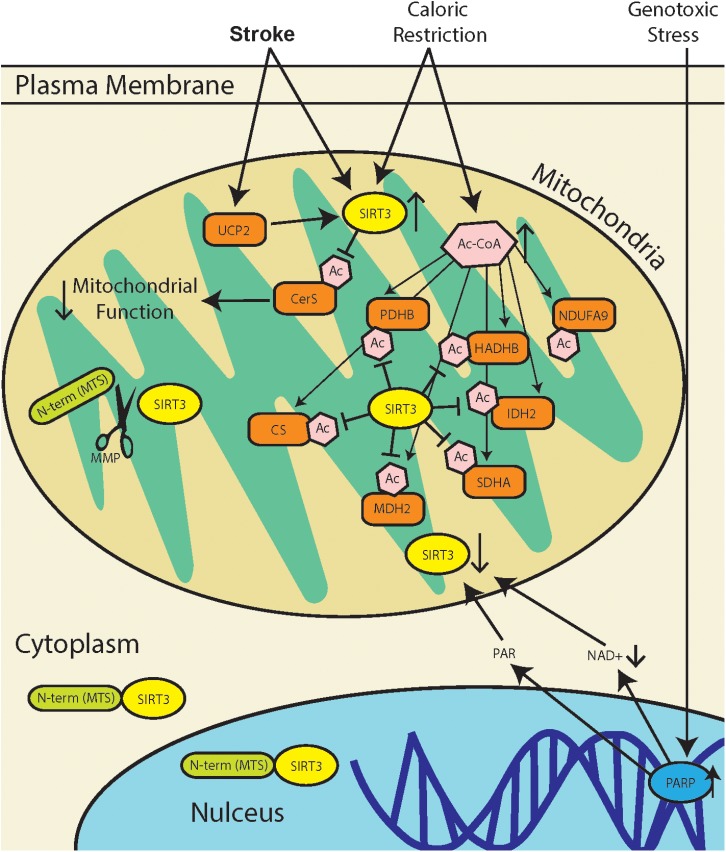
SIRT3 regulation during stress response. Stroke results in a combination of genotoxic, oxidative, and metabolic stresses, all of which have different effects on SIRT3 activity. Metabolic stress due to caloric restriction result in an increase in acetyl-CoA (Ac-CoA) which can acetylate proteins through non-enzymatic mechanisms (Weinert et al., 2015). SIRT3 can then repair acetylated mitochondrial proteins including pyruvate dehydrogenase (PDHB), citrate synthase (CS), acetyl-CoA acyltransferase (HADHB), isocitrate dehydrogenase 2 (IDH2), NADH dehydrogenase alpha subcomplex subunit 9 (NDUFA9), malate dehydrogenase 2 (MDH2), and succinate dehydrogenase complex subunit A (SDHA). Genotoxic stress, which occurs after stroke, leads to poly(ADP-ribose) polymerase (PARP) hyperactivation. This leads to a decrease in [NAD+], as well as accumulation of poly(ADP-ribose) polymer (PAR), and this may lead to downregulation of SIRT3. Mitochondria dysfunction in stroke can increase expression of mitochondrial uncoupling protein 2 (UCP2), which decreases ATP production and increases mitochondrial NAD+/NADH ratio, thereby increasing SIRT3 activity. Recent reports of SIRT3 activity in stroke demonstrate that SIRT3 deacetylates and activates ceramide synthases (CerS) which results in mitochondrial dysfunction.

The regulation of protein acetylation as a post-translational modification, orchestrated in mitochondria by SIRT3, has diverse functional consequences (summarized in Figure 1). Heritable polymorphisms in the promoter region of SIRT3, increasing its expression is correlated with longevity in humans (Albani et al., 2014). Yet, a recent study demonstrates that SIRT3 activity in stroke increases mitochondrial dysfunction (Tseng et al., 2014). These may seem like conflicting perspectives, but the literature demonstrates that there are tissue specific differences in SIRT3 regulation, as well as tissue specific sensitivities to increased or decreased activity. The overarching function of SIRT3 is to maintain mitochondrial homeostasis, and this can be achieved through modulating the activity of different mitochondrial proteins depending the tissue type. Adipose tissue that produce thermal energy, have a different mitochondrial environment as skeletal muscle that produce high concentrations of ATP.

Despite numerous studies, it is unclear how SIRT3 is being regulated in various tissues and model systems, and under various forms of other cellular stresses. SIRT3 is upregulated under conditions of nutrient deprivation, and under sustained nutrient deprivation, levels gradually decrease over time (Hirschey et al., 2011). This conditioning effect has not been observed *in vitro*, suggesting that multiple organ systems and a complex tissue environment is required for this metabolic shift. Conversely, investigators found that excess nutrient or high fat diet leads to down regulation of SIRT3. Nutrient deprivation may damage mitochondrial proteins through a decrease in rate of metabolite turnover. One hypothesis states that mitochondrial proteins become acetylated from an increased pool of mitochondrial acetyl-CoA. SIRT3 is then upregulated in response to increase Acetyl-lysine stoichiometry and repairs damaged proteins, restoring their function. Cells may then utilize lipid metabolism as an energy source, further increasing mitochondrial acetyl-CoA and sustaining SIRT3 up regulation as a positive feedback loop. Hypothesis such as these are testable and will enlighten a rapidly expanding and diverse body of literature, and although the literature may appear inconsistent and ambiguous, common trends highlighted here allow for more focused experimental design.

## Author Contributions

SA and JM reviewed the literature, planned and wrote the manuscript.

## Conflict of Interest Statement

The authors declare that the research was conducted in the absence of any commercial or financial relationships that could be construed as a potential conflict of interest.

## References

[B1] Ahn B. H. Kim H.-S. Song S. Lee I. H. Liu J. Vassilopoulos A. (2008). A role for the mitochondrial deacetylase Sirt3 in regulating energy homeostasis. *Proc. Natl. Acad. Sci. U.S.A.* 105 14447–14452. 10.1073/pnas.0803790105 18794531 PMC2567183

[B2] Albani D. Ateri E. Mazzuco S. Ghilardi A. Rodilossi S. Biella G. (2014). Modulation of human longevity by SIRT3 single nucleotide polymorphisms in the prospective study “Treviso Longeva (TRELONG)”. *Age* 36 469–478. 10.1007/s11357-013-9559-2 23839864 PMC3889902

[B3] Andrabi S. A. Kang H. C. Haince J. F. Lee Y. I. Zhang J. Chi Z. (2011). Iduna protects the brain from glutamate excitotoxicity and stroke by interfering with poly(ADP-ribose) polymer-induced cell death. *Nat. Med.* 17 692–699. 10.1038/nm.2387 21602803 PMC3709257

[B4] Andrabi S. A. Kim N. S. Yu S. W. Wang H. Koh D. W. Sasaki M. (2006). Poly(ADP-ribose) (PAR) polymer is a death signal. *Proc. Natl. Acad. Sci. U.S.A.* 103 18308–18313. 10.1073/pnas.0606526103 17116882 PMC1838747

[B5] Andrabi S. A. Umanah G. K. Chang C. Stevens D. A. Karuppagounder S. S. Gagné J. P. (2014). Poly(ADP-ribose) polymerase-dependent energy depletion occurs through inhibition of glycolysis. *Proc. Natl. Acad. Sci. U.S.A.* 111 10209–10214. 10.1073/pnas.1405158111 24987120 PMC4104885

[B6] Baek S. H. Bae O. N. Kim E. K. Yu S. W. (2013). Induction of mitochondrial dysfunction by poly(ADP-ribose) polymer: implication for neuronal cell death. *Mol. Cells* 36 258–266. 10.1007/s10059-013-0172-0 23996529 PMC3887971

[B7] Barger J. L. Vann J. M. Cray N. L. Pugh T. D. Mastaloudis A. Hester S. N. (2017). Identification of tissue-specific transcriptional markers of caloric restriction in the mouse and their use to evaluate caloric restriction mimetics. *Aging Cell* 16 750–760. 10.1111/acel.12608 28556428 PMC5506434

[B8] Baxter P. Chen Y. Xu Y. Swanson R. A. (2014). Mitochondrial dysfunction induced by nuclear poly(ADP-ribose) polymerase-1: a treatable cause of cell death in stroke. *Transl. Stroke Res.* 5 136–144. 10.1007/s12975-013-0283-0 24323707 PMC4034530

[B9] Blacker T. S. Mann Z. F. Gale J. E. Ziegler M. Bain A. J. Szabadkai G. (2014). Separating NADH and NADPH fluorescence in live cells and tissues using FLIM. *Nat. Commun.* 5:3936. 10.1038/ncomms4936 24874098 PMC4046109

[B10] Calabrese V. Cornelius C. Cuzzocrea S. Iavicoli I. Rizzarelli E. Calabrese E. J. (2011). Hormesis, cellular stress response and vitagenes as critical determinants in aging and longevity. *Mol. Aspects Med.* 32 279–304. 10.1016/j.mam.2011.10.007 22020114

[B11] Calabrese V. Cornelius C. Dinkova-Kostova A. T. Calabrese E. J. Mattson M. P. (2010). Cellular stress responses, the hormesis paradigm, and vitagenes: novel targets for therapeutic intervention in neurodegenerative disorders. *Antioxid Redox Signal.* 13 1763–1811. 10.1089/ars.2009.3074 20446769 PMC2966482

[B12] Canto C. Sauve A. A. Bai P. (2013). Crosstalk between poly(ADP-ribose) polymerase and sirtuin enzymes. *Mol. Aspects Med.* 34 1168–1201. 10.1016/j.mam.2013.01.004 23357756 PMC3676863

[B13] Cao K. Lei X. Liu H. Zhao H. Guo J. Chen Y. (2017). Polydatin alleviated radiation-induced lung injury through activation of Sirt3 and inhibition of epithelial-mesenchymal transition. *J. Cell. Mol. Med.* 21 3264–3276. 10.1111/jcmm.13230 28609013 PMC5706589

[B14] Chen T. Dai S. H. Li X. Luo P. Zhu J. Wang Y. H. (2018). Sirt1-Sirt3 axis regulates human blood-brain barrier permeability in response to ischemia. *Redox Biol.* 14 229–236. 10.1016/j.redox.2017.09.016 28965081 PMC5633840

[B15] Dai S. H. Chen T. Li X. Yue K. Y. Luo P. Yang L. K. (2017). Sirt3 confers protection against neuronal ischemia by inducing autophagy: involvement of the AMPK-mTOR pathway. *Free Radic Biol. Med.* 108 345–353. 10.1016/j.freeradbiomed.2017.04.005 28396174

[B16] Edgett B. A. Hughes M. C. Matusiak J. B. Perry C. G. Simpson C. A. Gurd B. J. (2016). SIRT3 gene expression but not SIRT3 subcellular localization is altered in response to fasting and exercise in human skeletal muscle. *Exp. Physiol.* 101 1101–1113. 10.1113/EP085744 27337034

[B17] Finkel T. Deng C. X. Mostoslavsky R. (2009). Recent progress in the biology and physiology of sirtuins. *Nature* 460 587–591. 10.1038/nature08197 19641587 PMC3727385

[B18] Frye R. A. (1999). Characterization of five human cDNAs with homology to the yeast SIR2 gene: sir2-like proteins (sirtuins) metabolize NAD and may have protein ADP-ribosyltransferase activity. *Biochem. Biophys. Res. Commun.* 260 273–279. 10.1006/bbrc.1999.0897 10381378

[B19] Frye R. A. (2000). Phylogenetic classification of prokaryotic and eukaryotic Sir2-like proteins. *Biochem. Biophys. Res. Commun.* 273 793–798. 10.1006/bbrc.2000.3000 10873683

[B20] Gagne J. P. Isabelle M. Lo K. S. Bourassa S. Hendzel M. J. Dawson V. L. (2008). Proteome-wide identification of poly(ADP-ribose) binding proteins and poly(ADP-ribose)-associated protein complexes. *Nucleic Acids Res.* 36 6959–6976. 10.1093/nar/gkn771 18981049 PMC2602769

[B21] Garcia-Fuentes E. Santiago-Fernández C. Gutiérrez-Repiso C. Mayas M. D. Oliva-Olivera W. Coín-Aragüez L. (2015). Hypoxia is associated with a lower expression of genes involved in lipogenesis in visceral adipose tissue. *J. Transl. Med.* 13:373. 10.1186/s12967-015-0732-5 26619907 PMC4663723

[B22] Guarente L. (2007). Sirtuins in aging and disease. *Cold Spring Harb Symp. Quant. Biol.* 72 483–488. 10.1101/sqb.2007.72.024 18419308

[B23] Gudiksen A. Pilegaard H. (2017). PGC-1alpha and fasting-induced PDH regulation in mouse skeletal muscle. *Physiol. Rep.* 5:e13222. 10.14814/phy2.13222 28400503 PMC5392513

[B24] Hallows W. C. Albaugh B. N. Denu J. M. (2008). Where in the cell is SIRT3?–functional localization of an NAD + -dependent protein deacetylase. *Biochem. J.* 411 e11–e13. 10.1042/BJ20080336 18363549 PMC3313448

[B25] Hallows W. C. Lee S. Denu J. M. (2006). Sirtuins deacetylate and activate mammalian acetyl-CoA synthetases. *Proc. Natl. Acad. Sci. U.S.A.* 103 10230–10235. 10.1073/pnas.0604392103 16790548 PMC1480596

[B26] Hebert A. S. Dittenhafer-Reed K. E. Yu W. Bailey D. J. Selen E. S. Boersma M. D. (2013). Calorie restriction and SIRT3 trigger global reprogramming of the mitochondrial protein acetylome. *Mol. Cell* 49 186–199. 10.1016/j.molcel.2012.10.024 23201123 PMC3704155

[B27] Hirschey M. D. Shimazu T. Goetzman E. Jing E. Schwer B. Lombard D. B. (2010). SIRT3 regulates mitochondrial fatty-acid oxidation by reversible enzyme deacetylation. *Nature* 464 121–125. 10.1038/nature08778 20203611 PMC2841477

[B28] Hirschey M. D. Shimazu T. Jing E. Grueter C. A. Collins A. M. Aouizerat B. (2011). SIRT3 deficiency and mitochondrial protein hyperacetylation accelerate the development of the metabolic syndrome. *Mol. Cell* 44 177–190. 10.1016/j.molcel.2011.07.019 21856199 PMC3563434

[B29] Huang J. Y. Hirschey M. D. Shimazu T. Ho L. Verdin E. (2010). Mitochondrial sirtuins. *Biochim. Biophys. Acta* 1804 1645–1651. 10.1016/j.bbapap.2009.12.021 20060508

[B30] Hung Y. P. Albeck J. G. Tantama M. Yellen G. (2011). Imaging cytosolic NADH-NAD( + ) redox state with a genetically encoded fluorescent biosensor. *Cell Metab.* 14 545–554. 10.1016/j.cmet.2011.08.012 21982714 PMC3190165

[B31] Iwahara T. Bonasio R. Narendra V. Reinberg D. (2012). SIRT3 functions in the nucleus in the control of stress-related gene expression. *Mol. Cell. Biol.* 32 5022–5034. 10.1128/MCB.00822-12 23045395 PMC3510539

[B32] Jablonski R. P. Kim S. J. Cheresh P. Williams D. B. Morales-Nebreda L. Cheng Y. (2017). SIRT3 deficiency promotes lung fibrosis by augmenting alveolar epithelial cell mitochondrial DNA damage and apoptosis. *FASEB J.* 31 2520–2532. 10.1096/fj.201601077R 28258190 PMC5434657

[B33] Jin L. Galonek H. Israelian K. Choy W. Morrison M. Xia Y. (2009). Biochemical characterization, localization, and tissue distribution of the longer form of mouse SIRT3. *Protein Sci.* 18 514–525. 10.1002/pro.50 19241369 PMC2760358

[B34] Jing E. Emanuelli B. Hirschey M. D. Boucher J. Lee K. Y. Lombard D. (2011). Sirtuin-3 (Sirt3) regulates skeletal muscle metabolism and insulin signaling via altered mitochondrial oxidation and reactive oxygen species production. *Proc. Natl. Acad. Sci. U.S.A.* 108 14608–14613. 10.1073/pnas.1111308108 21873205 PMC3167496

[B35] Jing E. O’Neill B. T. Rardin M. J. Kleinridders A. Ilkeyeva O. R. Ussar S. (2013). Sirt3 regulates metabolic flexibility of skeletal muscle through reversible enzymatic deacetylation. *Diabetes* 62 3404–3417. 10.2337/db12-1650 23835326 PMC3781465

[B36] Kang H. C. Lee Y. I. Shin J. H. Andrabi S. A. Chi Z. Gagné J. P. (2011). Iduna is a poly(ADP-ribose) (PAR)-dependent E3 ubiquitin ligase that regulates DNA damage. *Proc. Natl. Acad. Sci. U.S.A.* 108 14103–14108. 10.1073/pnas.1108799108 21825151 PMC3161609

[B37] Komjati K. Besson V. C. Szabo C. (2005). Poly (adp-ribose) polymerase inhibitors as potential therapeutic agents in stroke and neurotrauma. *Curr. Drug Targets CNS Neurol. Disord.* 4 179–194. 10.2174/1568007053544138 15857303

[B38] Leung A. K. (2014). Poly(ADP-ribose): an organizer of cellular architecture. *J. Cell. Biol.* 205 613–619. 10.1083/jcb.201402114 24914234 PMC4050725

[B39] Li L. Zhang P. Bao Z. Wang T. Liu S. Huang F. (2016). PGC-1alpha Promotes ureagenesis in mouse periportal hepatocytes through SIRT3 and SIRT5 in response to glucagon. *Sci. Rep.* 6:24156. 10.1038/srep24156 27052737 PMC4823758

[B40] Liu P. Zou D. Chen K. Zhou Q. Gao Y. Huang Y. (2016). Dihydromyricetin improves hypobaric hypoxia-induced memory impairment via modulation of sirt3 signaling. *Mol. Neurobiol.* 53 7200–7212. 10.1007/s12035-015-9627-y 26687185

[B41] Liu R. Fan M. Candas D. Qin L. Zhang X. Eldridge A. (2015). CDK1-Mediated SIRT3 activation enhances mitochondrial function and tumor radioresistance. *Mol. Cancer Ther.* 14 2090–2102. 10.1158/1535-7163.MCT-15-0017 26141949 PMC4560959

[B42] Liu S. G. Wang Y. M. Zhang Y. J. He X. J. Ma T. Song W. (2017). ZL006 protects spinal cord neurons against ischemia-induced oxidative stress through AMPK-PGC-1alpha-Sirt3 pathway. *Neurochem. Int.* 108 230–237. 10.1016/j.neuint.2017.04.005 28411102

[B43] Lombard D. B. Alt F. W. Cheng H. L. Bunkenborg J. Streeper R. S. Mostoslavsky R. (2007). Mammalian Sir2 homolog SIRT3 regulates global mitochondrial lysine acetylation. *Mol. Cell. Biol.* 27 8807–8814. 10.1128/MCB.01636-07 17923681 PMC2169418

[B44] Michishita E. Park J. Y. Burneskis J. M. Barrett J. C. Horikawa I. (2005). Evolutionarily conserved and nonconserved cellular localizations and functions of human SIRT proteins. *Mol. Biol. Cell.* 16 4623–4635. 10.1091/mbc.e05-01-0033 16079181 PMC1237069

[B45] Morigi M. Perico L. Rota C. Longaretti L. Conti S. Rottoli D. (2015). Sirtuin 3-dependent mitochondrial dynamic improvements protect against acute kidney injury. *J. Clin. Invest.* 125 715–726. 10.1172/JCI77632 25607838 PMC4319434

[B46] Novgorodov S. A. Gudz T. I. (2011). Ceramide and mitochondria in ischemic brain injury. *Int. J. Biochem. Mol. Biol.* 2 347–361. 22187669 PMC3242427

[B47] Novgorodov S. A. Riley C. L. Keffler J. A. Yu J. Kindy M. S. Macklin W. B. (2016). SIRT3 deacetylates ceramide synthases: implications for mitochondrial dysfunction and brain injury. *J. Biol. Chem.* 291 1957–1973. 10.1074/jbc.M115.668228 26620563 PMC4722471

[B48] Pillai V. B. Bindu S. Sharp W. Fang Y. H. Kim G. Gupta M. (2016). Sirt3 protects mitochondrial DNA damage and blocks the development of doxorubicin-induced cardiomyopathy in mice. *Am. J. Physiol. Heart Circ. Physiol.* 310 H962–H972. 10.1152/ajpheart.00832.2015 26873966 PMC4867337

[B49] Pillai V. B. Kanwal A. Fang Y. H. Sharp W. W. Samant S. Arbiser J. (2017). Honokiol, an activator of Sirtuin-3 (SIRT3) preserves mitochondria and protects the heart from doxorubicin-induced cardiomyopathy in mice. *Oncotarget* 8 34082–34098. 10.18632/oncotarget.16133 28423723 PMC5470953

[B50] Qiao A. Wang K. Yuan Y. Guan Y. Ren X. Li L. (2016). Sirt3-mediated mitophagy protects tumor cells against apoptosis under hypoxia. *Oncotarget* 7 43390–43400. 10.18632/oncotarget.9717 27270321 PMC5190031

[B51] Revollo J. R. Grimm A. A. Imai S. (2004). The NAD biosynthesis pathway mediated by nicotinamide phosphoribosyltransferase regulates Sir2 activity in mammalian cells. *J. Biol. Chem.* 279 50754–50763. 10.1074/jbc.M408388200 15381699

[B52] Satterstrom F. K. Swindell W. R. Laurent G. Vyas S. Bulyk M. L. Haigis M. C. (2015). Nuclear respiratory factor 2 induces SIRT3 expression. *Aging Cell* 14 818–825. 10.1111/acel.12360 26109058 PMC4568969

[B53] Scher M. B. Vaquero A. Reinberg D. (2007). SirT3 is a nuclear NAD + -dependent histone deacetylase that translocates to the mitochondria upon cellular stress. *Genes Dev.* 21 920–928. 10.1101/gad.1527307 17437997 PMC1847710

[B54] Schlicker C. Gertz M. Papatheodorou P. Kachholz B. Becker C. F. Steegborn C. (2008). Substrates and regulation mechanisms for the human mitochondrial sirtuins Sirt3 and Sirt5. *J. Mol. Biol.* 382 790–801. 10.1016/j.jmb.2008.07.048 18680753

[B55] Schwer B. Bunkenborg J. Verdin R. O. Andersen J. S. Verdin E. (2006). Reversible lysine acetylation controls the activity of the mitochondrial enzyme acetyl-CoA synthetase 2. *Proc. Natl. Acad. Sci. U.S.A.* 103 10224–10229. 10.1073/pnas.0603968103 16788062 PMC1502439

[B56] Schwer B. North B. J. Frye R. A. Ott M. Verdin E. (2002). The human silent information regulator (Sir)2 homologue hSIRT3 is a mitochondrial nicotinamide adenine dinucleotide-dependent deacetylase. *J. Cell. Biol.* 158 647–657. 10.1083/jcb.200205057 12186850 PMC2174009

[B57] Shi T. Wang F. Stieren E. Tong Q. (2005). SIRT3, a mitochondrial sirtuin deacetylase, regulates mitochondrial function and thermogenesis in brown adipocytes. *J. Biol. Chem.* 280 13560–13567. 10.1074/jbc.M414670200 15653680

[B58] Sol E. M. Wagner S. A. Weinert B. T. Kumar A. Kim H. S. Deng C. X. (2012). Proteomic investigations of lysine acetylation identify diverse substrates of mitochondrial deacetylase sirt3. *PLoS One* 7:e50545. 10.1371/journal.pone.0050545 23236377 PMC3517600

[B59] Someya S. Yu W. Hallows W. C. Xu J. Vann J. M. Leeuwenburgh C. (2010). Sirt3 mediates reduction of oxidative damage and prevention of age-related hearing loss under caloric restriction. *Cell* 143 802–812. 10.1016/j.cell.2010.10.002 21094524 PMC3018849

[B60] Stein L. R. Imai S. (2012). The dynamic regulation of NAD metabolism in mitochondria. *Trends Endocrinol. Metab.* 23 420–428. 10.1016/j.tem.2012.06.005 22819213 PMC3683958

[B61] Su J. Liu J. Yan X. Y. Zhang Y. Zhang J. J. Zhang L. C. (2017). Cytoprotective effect of the UCP2-SIRT3 signaling pathway by decreasing mitochondrial oxidative stress on cerebral ischemia-reperfusion injury. *Int. J. Mol. Sci.* 18:E1599. 10.3390/ijms18071599 28737710 PMC5536086

[B62] Sundaresan N. R. Samant S. A. Pillai V. B. Rajamohan S. B. Gupta M. P. (2008). SIRT3 is a stress-responsive deacetylase in cardiomyocytes that protects cells from stress-mediated cell death by deacetylation of Ku70. *Mol. Cell. Biol.* 28 6384–6401. 10.1128/MCB.00426-08 18710944 PMC2577434

[B63] Taguchi K. Motohashi H. Yamamoto M. (2011). Molecular mechanisms of the Keap1-Nrf2 pathway in stress response and cancer evolution. *Genes Cells* 16 123–140. 10.1111/j.1365-2443.2010.01473.x 21251164

[B64] Tao N. N. Zhou H. Z. Tang H. Cai X. F. Zhang W. L. Ren J. H. (2016). Sirtuin 3 enhanced drug sensitivity of human hepatoma cells through glutathione S-transferase pi 1/JNK signaling pathway. *Oncotarget* 7 50117–50130. 10.18632/oncotarget.10319 27367026 PMC5226572

[B65] Tseng A. H. Wu L. H. Shieh S. S. Wang D. L. (2014). SIRT3 interactions with FOXO3 acetylation, phosphorylation and ubiquitinylation mediate endothelial cell responses to hypoxia. *Biochem. J.* 464 157–168. 10.1042/BJ20140213 25162939

[B66] Ugur S. Ulu R. Dogukan A. Gurel A. Yigit I. P. Gozel N. (2015). The renoprotective effect of curcumin in cisplatin-induced nephrotoxicity. *Ren. Fail.* 37 332–336. 10.3109/0886022X.2014.986005 25594614

[B67] VanLinden M. R. Dölle C. Pettersen I. K. Kulikova V. A. Niere M. Agrimi G. (2015). Subcellular distribution of NAD + between cytosol and mitochondria determines the metabolic profile of human cells. *J. Biol. Chem.* 290 27644–27659. 10.1074/jbc.M115.654129 26432643 PMC4646015

[B68] Verma R. Ritzel R. M. Crapser J. Friedler B. D. McCullough L. D. (2018). Evaluation of the neuroprotective effect of Sirt3 in experimental stroke. *Transl. Stroke Res.* 10.1007/s12975-017-0603-x [Epub ahead of print]. 29302794 PMC9527822

[B69] Wang Q. Li L. Li C. Y. Pei Z. Zhou M. Li N. (2015). SIRT3 protects cells from hypoxia via PGC-1alpha- and MnSOD-dependent pathways. *Neuroscience* 286 109–121. 10.1016/j.neuroscience.2014.11.045 25433241

[B70] Wang S. Zhang C. Niyazi S. Zheng L. Li J. Zhang W. (2017). A novel cytoprotective peptide protects mesenchymal stem cells against mitochondrial dysfunction and apoptosis induced by starvation via Nrf2/Sirt3/FoxO3a pathway. *J. Transl. Med.* 15:33. 10.1186/s12967-017-1144-5 28202079 PMC5309997

[B71] Wang X. Tomso D. J. Chorley B. N. Cho H. Y. Cheung V. G. Kleeberger S. R. (2007). Identification of polymorphic antioxidant response elements in the human genome. *Hum. Mol. Genet.* 16 1188–1200. 10.1093/hmg/ddm066 17409198 PMC2805149

[B72] Weinert B. T. Moustafa T. Iesmantavicius V. Zechner R. Choudhary C. (2015). Analysis of acetylation stoichiometry suggests that SIRT3 repairs nonenzymatic acetylation lesions. *EMBO J.* 34 2620–2632. 10.15252/embj.201591271 26358839 PMC4641529

[B73] Yang F. Zhou L. Wang D. Wang Z. Huang Q. Y. (2015). Minocycline ameliorates hypoxia-induced blood-brain barrier damage by inhibition of HIF-1alpha through SIRT-3/PHD-2 degradation pathway. *Neuroscience* 304 250–259. 10.1016/j.neuroscience.2015.07.051 26211444

[B74] Yang W. Nagasawa K. Münch C. Xu Y. Satterstrom K. Jeong S. (2016). Mitochondrial sirtuin network reveals dynamic SIRT3-dependent deacetylation in response to membrane depolarization. *Cell* 167 985.e21–1000.e21. 10.1016/j.cell.2016.10.016 27881304 PMC5134900

[B75] Yang X. Geng K. Zhang J. Zhang Y. Shao J. Xia W. (2017). Sirt3 mediates the inhibitory effect of adjudin on astrocyte activation and glial scar formation following ischemic stroke. *Front. Pharmacol.* 8:943. 10.3389/fphar.2017.00943 29311941 PMC5744009

[B76] Yin J. Han P. Tang Z. Liu Q. Shi J. (2015). Sirtuin 3 mediates neuroprotection of ketones against ischemic stroke. *J. Cereb. Blood Flow Metab.* 35 1783–1789. 10.1038/jcbfm.2015.123 26058697 PMC4635233

[B77] Yoon S. P. Kim J. (2016). Poly(ADP-ribose) polymerase 1 contributes to oxidative stress through downregulation of sirtuin 3 during cisplatin nephrotoxicity. *Anat. Cell Biol.* 49 165–176. 10.5115/acb.2016.49.3.165 27722009 PMC5052225

[B78] Yu S. W. Andrabi S. A. Wang H. Kim N. S. Poirier G. G. Dawson T. M. (2006). Apoptosis-inducing factor mediates poly(ADP-ribose) (PAR) polymer-induced cell death. *Proc. Natl. Acad. Sci. U.S.A.* 103 18314–18319. 10.1073/pnas.0606528103 17116881 PMC1838748

[B79] Zhang M. Zhao Z. Shen M. Zhang Y. Duan J. Guo Y. (2017). Polydatin protects cardiomyocytes against myocardial infarction injury by activating Sirt3. *Biochim. Biophys. Acta.* 1863 1962–1972. 10.1016/j.bbadis.2016.09.003 27613967

